# Influence of Abutment Screw-Tightening Methods on the Screw Joint: Immediate and Long-Term Stability

**DOI:** 10.1155/2024/5768318

**Published:** 2024-01-16

**Authors:** Manlin Sun, Yusen Shui, Yuqiang Zhang, Ruiyang Ma, Yuwei Zhao, Hongyu Chen, Ping Yu, Zhi Li, Tingting Wu, Haiyang Yu

**Affiliations:** State Key Laboratory of Oral Diseases, National Clinical Research Center for Oral Diseases and Department of Prosthodontics, West China Hospital of Stomatology, Sichuan University, Chengdu, China

## Abstract

**Objective:**

To evaluate the influence of screw-tightening methods on the immediate and long-term stability of dental implant screw joints. *Methodology*. A total of 150 implants of three different implant systems with different diameters were used in this study. Each group was divided into three subgroups (*n* = 5), according to the tightening methods (A—tightening with recommended torque and retorque after 10 min; B—tightening with recommended torque, then loosening and immediate retorque; C—tightening with recommended torque only once). The operating time of tightening the assemblies was recorded. Ten minutes later, the immediate removal torque (IRT) (Ncm) was measured. After retightening the assemblies, a dynamic load between 20 and 200 N was applied for 10^5^ cycles, and the postloading removal torque (PRT) (Ncm) was measured. Scanning electron microscopy (SEM) was used to observe the surface topography of the screws.

**Results:**

For different types of implants, the IRTs were 11.92 ± 1.04–34.12 ± 0.36 Ncm for method A, 11.64 ± 0.57–33.96 ± 0.29 Ncm for method B, and 10.30 ± 0.41–31.62 ± 0.52 Ncm for method C, and the IRTs of methods A and B were 6.28%–21.58% higher than that of method C (*P* ≤ 0.046). The PRTs were 4.08 ± 0.77–29.86 ± 0.65 Ncm for method A, 4.04 ± 0.40–29.60 ± 0.36 Ncm for method B, and 2.98 ± 0.26–26.38 ± 0.59 Ncm for method C, and the PRTs of methods A and B were 11.77%–44.87% higher than that of method C (*P* ≤ 0.016). The removal torque loss rates of methods A (12.49% ± 0.99%–65.88% ± 4.83%) and B (12.84% ± 0.96%–65.35% ± 1.95%) were 3.04%–7.74% lower than that of method C (16.58% ± 0.56%–71.10% ± 1.58%) (*P* ≤ 0.017). The operating time of method A was much longer than those of methods B and C (*P* < 0.001). The structural integrity disruption of the screw thread was observed according to the SEM results in all postloading groups.

**Conclusions:**

Method B (torquing and then loosening and immediate retorquing) increases the screw joint immediate stability by 6.28%–21.58% and the long-term stability by 11.77%–44.87% compared with method C (torquing only once), has comparable screw joint stability compared with method A (retorquing after 10 min), saves time and is recommended in clinical settings.

## 1. Introduction

Despite the recognized success of dental implants, long-term clinical studies of implant-supported prostheses have reported several complications, including mechanical complications, which exert adverse effects on long-term clinical performance. Among them, screw loosening is a major mechanical complication. A 14-year systematic review of single or two-unit implant fixed dental prostheses indicated a screw loosening rate ranging from 7% to 11% [1]. During the first year of use, loosening occurred in 26% of gold prosthesis-retaining screws and 43% of abutment screws [2]. Screw joint instability between the abutment and the implant may cause not only micromotion at the implant–abutment interface, inappropriate stress distribution, and fatigue failure of the screw but also an expansion of the microgap between the implant and the abutment, which may result in both screw loosening and an inflammatory reaction in peri-implant soft tissues, leading to long-term prosthesis complications [3–8]. In the clinic, screw loosening brings about an increase in subsequent visits for maintenance, which is time-consuming and inconvenient for patients and doctors. The loosening effect is more serious for cemented restoration due to the necessity to break the crown to gain access to the screw. Removal of the crown is a problem in many patients, which causes not only extra cost but also irritation and pain [9, 10]. Considering the adverse consequences mentioned above, research on improving screw joint stability is an important topic in the dental implant field.

Screw joint stability is associated with several factors, such as the shape of the abutment screw head [11, 12], material properties of the abutment screw [13–15], design of the implant–abutment interface [15–19], functional load, preload or clamping force, thread embedment relaxation [20], and thread shape and misfit [21]. The methods proposed to prevent loosening in order to clinically achieve screw joint stability have been designed primarily from the perspective of sufficient preload in recent years. Preload is the term given to the force within the screw when torque is applied, which mainly depends on the frictional coefficient of the screw [17, 22], the condition of lubricant (if used) [23], the speed of tightening, and the tightening force used [24]. The preload tightly and consistently secures the screw threads to the screw's mating pair. When the clamping force developed within the mating pair is less than the forces that pull the components apart, screw loosening occurs [24]. However, considerable preload in the screw of approximately 17%–29% may be lost [25]. Owing to the settling effect, which occurs when the screw-implant interface wear enables closer contact between the two surfaces, followed by decreasing of the tension or clamping force, the screw is more easily loosened [17, 25–27].

To minimize the adverse effect of settling on preload, some researchers suggested that abutment screws should be retightened after the initial torque applications [8, 25, 28, 29], as retorquing is reported to increase removal torque by 13.7%–32% [29] and decrease torque force loss by nearly 50% [8] compared with torquing only once. However, there are no details about specific tightening methods in the instructions of different implant systems, such as the interval time between two instances of tightening, and there is still no consensus in this regard. Siamos et al. [25] suggested that retightening 10 min after the initial tightening should be performed as a routine clinical procedure. However, this method was poorly received to some extent because it would increase the chairside time, decrease the comfort of patients and increase the possibility of saliva contamination of the screw channel. Researchers have thus focused on the issue of whether shortening the interval time would influence screw stability. A previous study revealed that variances in the initial removal torque of different groups with various interval times (2, 5, and 10 min) did not show significant differences [8]. Meanwhile, another study focused on a more time-economical method that involved tightening, countertightening, and immediate retightening. Neither this method nor retorquing with a 10-min interval time improved the initial removal torque for the Neoss implant system [30].

The different experimental results mentioned above may have occurred due to various experimental designs in the studies. The clinical success of implant restoration should be evaluated from a long-term perspective and in the context of *in vitro* simulation of long-term functional complex conditions to improve the meaning of clinical guidance. The most widely used method for simulating long-term functional conditions is “fatigue testing,” including cyclic loading [31], which had a significant effect on screw torque values within the implant–abutment system in an *in vitro* study [25, 28]. However, some of the studies [25, 32–34] performed cyclic loading, while others did not [8, 30]. Studies focusing on retorquing methods with a 10-min interval time involved several loading designs, including a 6-kg load for 16,660 cycles, which is equivalent to 1 month of intraoral loading [32]; 21,600 loading cycles with valves cycled between 1 and 26 pounds [25], loading at 9 Hz until failure or for the maximum of 250,000 cycles at 190 N [33]; and 500,000 cycles of loading between 0 and 200 N, equivalent to 6 months of intraoral loading [34]. These long-term postloading evaluations increase the validity of the results. Moreover, there was a lack of long-term postloading evaluations of tightening methods with shorter interval times. One study [30] measured the removal torque three hours after tightening without loading, while a different study [8] measured the removal torque 30 min after tightening, which rendered it difficult to assess the effect of shortening the interval time on the long-term screw joint stability. Furthermore, the majority of the studies [7, 25, 33, 34] focusing on the effects of different tightening methods on screw stability used implants of only one brand with only one diameter parameter, which limits the application of those conclusions to other implant systems or other implants with different diameters.

Thus, this *in vitro* study aimed to objectively evaluate the influence of different tightening methods on the removal torque value in different dental implant systems with various diameters before and after simulating oral cyclic loading. We hypothesized that the initial and long-term postloading removal torques (PRTs) of different tightening methods were not significantly different and that the preferable tightening methods for different implant systems and different-diameter implants were the same.

## 2. Methodology

### 2.1. Sample and Materials

The present study used three implant systems: the Nobel Replace Conical Connection implant (Nobel Biocare, Kloten, Switzerland), the Straumann Bone Level implant (Straumann, Basel, Switzerland), and the WEGO implant (WEGO, WeiHai, China). Same-length but different-diameter implants of each system were selected, and the lengths of implants were similar among the three systems. Each chosen diameter consisted of 15 implants in each system. The implants and abutment screws selected are presented in Table 1.

### 2.2. Torquing and Loading Procedures

**Figure 1:**
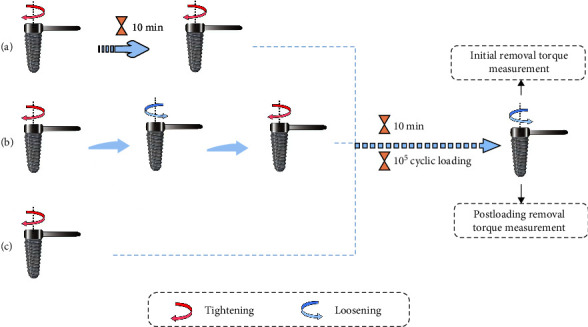
Experimental protocol of different groups: (a) tightening method A; (b) tightening method B; (c) tightening method C.

Same-diameter specimens from each system were embedded in polymethyl methacrylate resin and randomly divided into three subgroups (five assemblies per subgroup) to apply three different tightening methods, as shown in Figure 1. Using a digital torque meter (LuTronTQ-8800, Taiwan, China) with a precision of 0.1 Ncm and a screwdriver, the torque recommended by each manufacturer was applied to the abutments (35 Ncm for the Nobel and Straumann systems, 20 Ncm for the WEGO system).

The implant–abutment assemblies from each system were subjected to three different tightening methods as follows: method A—tightening with the recommended torque and retorque after 10 min; method B—tightening with the recommended torque, loosening and retorquing immediately with the same torque; and method C—tightening with the recommended torque only once.

First, the assemblies were tightened using each tightening method. 10 min later, the immediate removal torque was measured as the initial removal torque (IRT) using the same digital torque meter. The assemblies were retightened using each tightening method. Then, a cylindrical CAD/CAM zirconium oxide cap was located on the abutment, and the implant–abutment assemblies were fitted to the jig [11].

**Figure 2:**
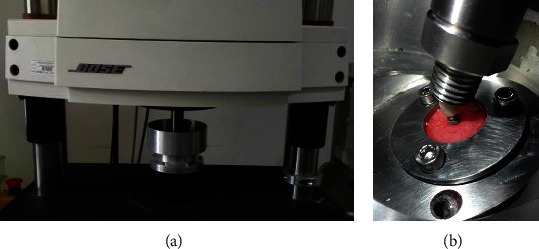
BioDynamic test instrument: (a) loading instrument; (b) the embedded implants under loading cycles.

The jig was installed in an ElectroForce3330 BioDynamic dynamic loading test instrument (WinTest 7 software, Bose, MN, USA) (Figure 2). A compressive cyclic sine wave load between 20 and 200 N at a loading frequency of 15 Hz was applied to the specimens for 10^5^ cycles. Then, the residual reverse torque values were recorded as the PRT. We calculated the removal lower torque loss rate (TLR) before and after loading with the following formula [27]:

(1) $$\begin{array}{c} \text{Torque loss rate }\left(\%\right)=\left(\text{Initial removal torque}-\text{postloading removal torque}\right)/\left(\text{Initial removal torque}\right)\times 100. \end{array}$$

The surface topography of an unused screw and screws after loading from each group were evaluated using SEM (Inspect F, Czech Republic).

### 2.3. Statistical Analysis

Statistical analysis was conducted using SPSS software (version 22.0, SPSS Inc., IL, USA). Data for each group were recorded. The normality and equal variance assumptions of the data were evaluated by Shapiro‒Wilk and Levene's tests. One-way analysis of variance (ANOVA) and Tukey's HSD test were performed for the statistical analysis of data with normal distribution and equal variance. If the data did not have a normal distribution and equal variance, a nonparametric one-way ANOVA (Kruskal‒Wallis) was performed. *P* values less than 0.05 indicated statistical significance.

According to the statistical analysis, the Kruskal–Wallis test was used in the following comparisons, including comparisons Nos. 38, 43, 45, 46, 47, 48, 52, 54, 56, 65, 74, and 76, as the data were not normally distributed or exhibited homogeneity of variance (Table 2). Further analyses were conducted using ANOVA tests.

## 3. Results

### 3.1. Comparison of Different Tightening Methods for Implants with the Same Diameter in Each System

**Figure 3:**
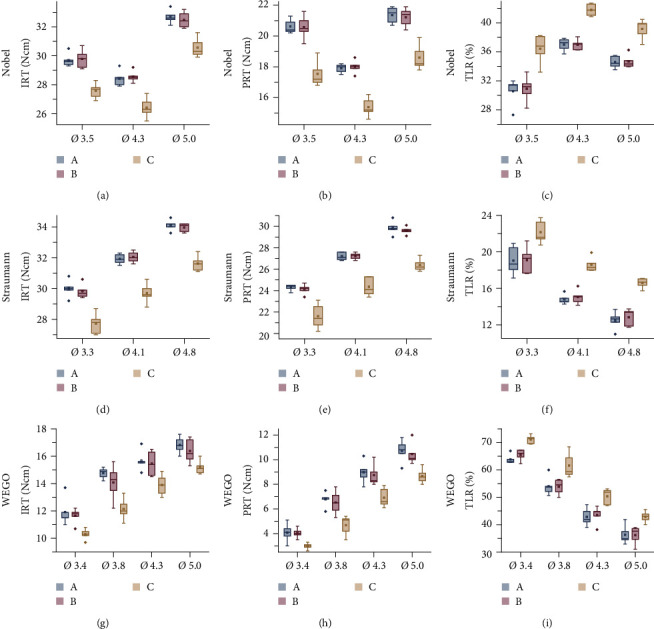
A comparison of initial removal torque (IRT) (Ncm), postloading removal torque (PRT) (Ncm) and removal torque loss rate (TLR) (%) of different implant diameter in each group: (a) IRT of Nobel system; (b) PRT of Nobel system; (c) TLR of Nobel system; (d) IRT of Straumann system; (e) PRT of Straumann system; (f) TLR of Straumann system; (g) IRT of WEGO system; (h) PRT of WEGO system; (i) TLR of WEGO system.

As shown in Table 1, in all subgroups, the IRTs of methods A and B were higher than those of method C, with significant differences in group Nobel (*P* ≤ 0.001), group Straumann (*P*  < 0.001), and group WEGO (*P* ≤ 0.046), and the PRTs of methods A and B were also higher than those of method C, with significant differences in group Nobel (*P*  < 0.001), group Straumann (*P*  < 0.001), and group WEGO (*P* ≤ 0.016). In most subgroups, the TLRs of methods A and B were lower than those of method C, with significant differences in the Nobel (*P* ≤ 0.002), Straumann (P ≤ 0.017), WEGO Ø3.8 (*P* ≤ 0.02), WEGO Ø4.3 (*P* ≤ 0.014), and WEGO Ø5.0 (*P* ≤ 0.014) groups, except for the WEGO Ø3.4 group, in which the TLRs of methods A and B were lower than those of method C without a significant difference. Between methods A and B, no significant difference was found in the IRTs (0.491 ≤ *P* ≤ 0.973), PRTs (0.739 ≤ *P* ≤ 0.998), or TLRs (0.805 ≤ *P* ≤ 1.000). The comparisons of IRT, PRT, and TRL in different groups are shown in Figure 3. For immediate stability evaluation, the IRTs of methods A, B, and C were 11.9–34.12 Ncm, 11.64–33.96 Ncm, and 10.30–31.62 Ncm, respectively. The IRTs of methods A and B were 6.28%−21.58% higher than that of method C (Table 1). For the long-term stability evaluation, the PRTs of methods A, B, and C were 4.08–29.86 Ncm, 4.04–29.60 Ncm, and 2.98–26.38 Ncm, respectively, and the PRTs of methods A and B were 11.77%−44.87% higher than that of method C. The TLRs of methods A, B, and C were 12.49%−65.88%, 12.84%−65.35%, and 16.58%−71.10%, respectively, and the TLRs of methods A and B were 3.04%–7.74% lower than that of method C. The removal torque loss and the differences (%) in IRT, PRT, and TLR between A and C and between B and C are also shown in Table 1.

### 3.2. Comparison of Different-Diameter Implants Using the Same Tightening Method in Each System

For the Nobel implant system, the IRT and the PRT with the same tightening method were the highest in Ø5.0, followed by Ø3.5 and Ø4.3, as shown in Figures 3(a) and 3(b). The IRT showed statistically significant differences between the different diameter groups in methods A (*P* ≤ 0.004), B (*P* ≤ 0.012), and C (*P* ≤ 0.04) (Table 1). For PRT, no difference was found between Ø3.5 and Ø5.0 in methods A (*P*=0.053), B (*P=0.276*), and C (*P*=0.123), while Ø4.3 exhibited significant differences in the PRT compared with the other two groups (*P* ≤ 0.002). The TLR was the highest in Ø4.3, followed by Ø5.0 and Ø3.5, as shown in Figure 3(c). When using the same tightening method, statistically significant differences in TLR were found for each tightening method, including methods A (*P* ≤ 0.038), B (*P* ≤ 0.034), and C (*P* ≤ 0.044) (Table 1).

For the Straumann implant system, the IRT and the PRT of Ø4.8 using the same tightening method were the highest, followed by Ø4.1 and Ø3.3, as shown in Figures 3(d) and 3(e). The TLR of Ø3.3 was the highest, followed by Ø4.1 and Ø4.8, as shown in Figure 3(f). The differences in IRT, PRT, and TLR were statistically significant for the same tightening methods between different diameter groups (*P* ≤ 0.023) (Table 1).

In the WEGO implant system, the IRT and PRT were highest in Ø5.0 using the same tightening method, followed by Ø4.3, Ø3.8, and Ø3.4, as shown in Figures 3(g) and 3(h) (Table 1). The TLR was the highest in Ø3.4 of WEGO, followed by Ø3.8, Ø4.3, and Ø5.0 (Table 1), as shown in Figure 3(i).

### 3.3. Comparison of the Operating Times of Different Tightening Methods

The operating times of the three tightening methods showed significant differences in each system (*P*  < 0.001) (Table 3). The operating time of tightening method A was more than 10 min, while the operating times of tightening methods B and C were less than 30 s.

### 3.4. Surface Topography of Abutment Screws

**Figure 4:**
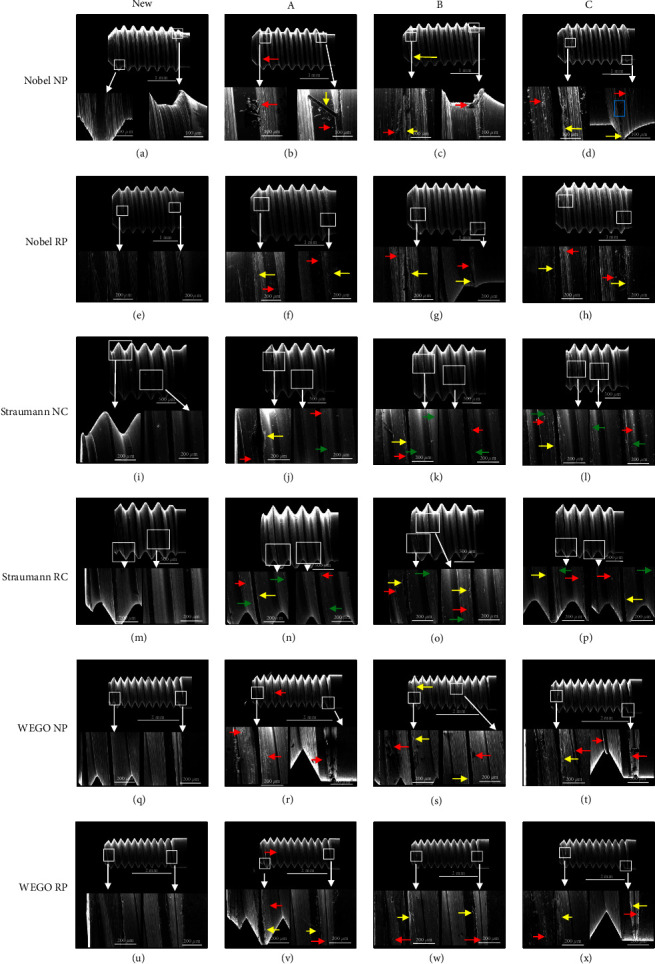
(a)–(x) SEM pictures of different abutment screws: each line of this figure shows SEM pictures of four different screws for a specific implant type. The first picture in each line shows a new unused screw, the second picture shows a screw after using tightening method A, the third picture shows a screw after using tightening method B, and the last picture shows a screw after using tightening method C. Red arrow indicates debris, yellow arrow indicates peeling, green arrow indicates scratch, and blue box indicates screw thread where screw material has peeled off.

Scanning electron microscopy (SEM) micrographs of the selected abutment screws are presented in Figure 4. The new screw surfaces were found to be homogeneous and nonporous with no surface debris. The screw thread morphology varied as the study progressed. The screw thread structural integrity was disrupted, primarily through peeling and debris. Different screw-tightening methods manifested a similar destructive mode for the abutment screws in each system.

Figure 4(a) shows that the new abutment screws in Nobel implants were striated. Debris was observed on the thread surface after loading (Figure 4(b)–4(d)). The screws had nonhomogeneous surfaces. The thread structural integrity was partly destroyed, but the continuity of the striated structure was not damaged. The wear debris and larger material debris were dispersed or gathered into a mass on the screw thread. A striped screw material, approximately 100–200 *µ*m long, was first peeled off from the Nobel screw thread, surrounded by wear debris (Figures 4(b) and 4(f)–4(h)). As the screw material peeled off, the thread crest presented a rugged surface after loading cycles (Figure 4(d)). Additionally, a thin smog-like screw material flaked off from the crest of the screw thread, with wear debris around it (Figures 4(c), 4(d), 4(g), and 4(h)).

However, Figures 4(i) and 4(m) show that the new Straumann screws were not striated. The scratches on Straumann screws were noticeable, appearing near the crest of the thread and not at the root of the thread, and were caused by sliding friction between the abutment screw threads and the internal threads of implants (Figures 4(j)–4(l) and 4(n)–4(p)). Some wear debris was distributed at the scratches. Straumann screws did not display peeling of long, striped material from the beginning of the screw thread, but a thin smog-like screw material flaked off from the crest of the screw thread.

The changes in WEGO screws were similar to those observed in Nobel screws. The new abutment screws of WEGO were striated (Figures 4(q) and 4(u)). Debris was present on the thread surface of the loaded screws, especially on the tail region (Figures 4(r)–4(t) and 4(v)–4(x)). The wear debris was observed to be dispersed or gathered into a mass near the crest of the screw thread (Figure 4(r)). Striped and lumpish debris (approximately 100–200 *μ*m long) adhered to the thread surface (Figures 4(s) and 4(t)). Approximately 200-*μ*m-long striped material was peeled off from the beginning of the WEGO screw thread, with small pieces of debris (Figure 4(v)). A thin smog-like flake screw material (20–40 *µ*m wide) was peeled off from the crest of the screw thread, with wear debris around it (Figures 4(s), 4(t), 4(w), and 4(x)).

## 4. Discussion

Abutment screw loosening is the most frequent complication of implant–abutment assemblies, and screw joint instability may cause serious consequences. Screw loosening prevention represents an important issue in the field. To gain improvement in screw stability from the perspective of clinical operations, this study evaluated the influence of screw-tightening methods on preload maintenance in different-diameter implants before and after cyclic loading application by measuring the removal torque and calculating the removal TLR.

The removal torque measurement of abutment screws is one of the methods for indirect comparison of preloads. The reduction degree of the preload could be compared after measuring the initial removal torque and the PRT. This study was based on the assumption of three episodes of chewing per day by an individual, each 15 min in duration, at a chewing rate of 60 cycles per minute (1 Hz). This is equivalent to 2,700 chewing cycles per day or approximately 10^5^ cycles per month [31, 35]. The stress state of the implants in the oral cavity is complex, so we set the loading force of the implant in a range of 20–200 N in the form of a sine wave, according to a previous study [31]. The results in the present study indicate that joint stability against the functional load is more critical in the clinic than the results of previous studies [8, 30]. Although the comparison of the removal torque of methods A, B, and C demonstrated similar IRT and PRT, TLR is still a useful index for evaluating screw stability, something that was lacking in previous study results [30].

This study hypothesized that the PRTs of different tightening methods would not be significantly different and that the preferable tightening methods for different implant systems and different-diameter implants would be the same. Our results demonstrated that the postloading torques of different tightening methods were significantly different; however, while also demonstrating that the preferable tightening methods for different implant systems were indeed the same, tightening methods A and B presented higher IRT and PRT and lower TLR than tightening method C in all groups, while no significant difference was found between tightening methods A and B. Tzenakis et al. [36] indicated that a higher preload is achieved after the repeated torque of an abutment screw, possibly owing to the reduced friction between the implant–abutment assemblies. A similar result was obtained in this study. However, the operating time of method A required more than 10 min, and methods B and C required less than 30 s. With limited clinical operating time, doctors would like to choose a time-saving and effective method. The present study recommended tightening method B for this purpose.

Similar to the results of this study, Farinaet et al. [29] also observed that retorque application resulted in significantly greater loosening torque of abutment screws of implant-supported fixed bridges after mechanical loads, and retorque application significantly improved the loosening torque in both the “passive fit” group and the “misfit” group. However, another study revealed that although retightening screws 10 min after the initial torque significantly increased the loosening torque in the “passive fit” group, it did not necessarily lead to better preload in a nonpassively fitting framework [21]. The difference in those results for the “misfit” group may be due to the use of different experimental models in these two studies. One of the studies involved implants in the model. In the other study, the abutments were directly fixed in the epoxy resin and were not connected with the implant, which could affect stress conduction. Furthermore, the other details about the experimental method, including the number of abutments in each framework, the implant system, the misfit design and the cyclic load, were also different between these two studies, which may lead to the contrasting conclusion. Further studies focusing on the effect of the retightening method on the stability of misfit screws are still needed.

To explain the mechanism of retightening in our study, there was a hypothesis that 2%–10% of the initial preload is lost on account of the settling effect [2]. In terms of tribology, when torquing a screw, the energy is expended in smoothing surface irregularities to maintain the assembly connectivity. The rough surface is flattened after thread engagement, and the additional tightening torque is applied toward the extension of the screw and induction of preload. Though the rough surface is flattened, the coefficient of the implant–abutment–screw complex may still increase. Previous studies [37, 38] revealed that retightening the abutment screw appeared to increase the coefficient of the screw and decrease the preload. Meanwhile, in mechanical engineering [39], it is suggested that the self-loosening of an assembled bolted joint over a period of time when subjected to fluctuating loads can be controlled by proper preload and thread friction. Thus, preload and thread friction both have a great effect on the stability of the dental implant screw joints. For the aspect of controlling the self-loosening effect, we assumed that after retightening, the friction debris still remained between the contact surfaces, resulting in the increase of the coefficient of friction and compensation of the negative influence of the reduction of the preload on the connection stability, thus controlled self-loosening. Moreover, in addition to the thread changes mentioned in this paper, it is also noted in the literatures [37, 38] that the preload in dental implants decreases with repeated tightening and that this is caused solely by the increase in friction on the screw head. Therefore, for the long-term stability of implant connections, further studies may focus on how the screw head effect on the balance of preload and the friction.

Moreover, various implant brands, as well as different-diameter implants, have recently been developed for specific clinical situations. The choice of implant diameter in the clinic depends on the edentulism type, the volume of the residual bone, the type of occlusion, the emergence profile, and the amount of space available for prosthetic reconstruction [40, 41]. Previous studies have failed to provide sufficient evidence regarding whether it was appropriate to apply the recommended tightening method to implants of different brands and different diameters [7, 25, 33, 34]. Different-diameter implants from three different implant systems were chosen in this study as samples, including two international common implant systems and a novel Chinese implant system. Based on the limited results of this study, including IRT, PRT, and TLR, the recommended method of tightening with the recommended torque and then loosening and retorquing immediately with the same torque can be applied for different-diameter implants.

In addition, it was found that when the size of abutment screws was the same, the wider-diameter implant–abutment assemblies showed higher IRT and PRT but lower TLR than the narrower assemblies. These results were in agreement with the findings of Reinhold et al. [42], who found that wide-diameter implants exhibited higher initial removal values and removal values after loading than regular implants. These results were also compatible with the findings of Lee et al. [43], who found that Group 5.0 mm exhibited significantly lower axial displacement and reverse torque loss after the cyclic and static loading of the overload condition than Group 4.0 mm and Group 4.5 mm of internal conical-design implant-abutment assembly. Another study also concluded that the screw loosening process occurred with higher values in narrow-diameter implants [28]. This result may be due to the wedge effect and the different thicknesses of implants with different diameters [43]. The wedge effect may lead to the deformation of the implant and diametric expansion under cyclic load. When the size of abutment screws is the same, a wider implant corresponds with a thicker lateral wall of the implant, and the wedge effect could be limited strictly by the thicker lateral wall of an implant with a larger diameter.

Interestingly, this study showed that the narrowest implant does not always present the smallest IRT and PRT and the largest TLR. Different from Straumann and WEGO, the IRT and PRT of the Nobel implant of group Ø3.5 were higher than those of group Ø4.3, while the TLR was lower than that of group Ø4.3. The reason for this result might be that the different Nobel abutment screws matched different designs (Figure 3). Although an NP Nobel abutment screw is thinner than an RP abutment screw, it has one more screw thread than the regular screw (Figure 3(a)–3(h)). It follows that the design details of the abutment screw play a crucial role in screw loosening. Screw stability is usually affected by many factors of the screw itself [44]: screw head design [11], thread design and number, type of metal [13, 14], surface condition [16, 18, 19], and length and diameter of the screw [23].

Strips of the screw material peeled off from the first threads of the abutment screw (Figures 4(a)–4(h), 4(q)–4(x)). According to previous FEA studies, the highest von Mises stress was distributed at the bottom of the initial two threads of the abutment screw [45, 46]. The reason underlying the results of this study may be that with a wider diameter, the stress of the interface was lower, so the friction of the initial two threads of the abutment screw decreased. Fewer strips peeled off, reducing the deformation of the materials. Thus, the implant complex gained screw joint stability.

However, there were still some limitations in this study. The sample size was based on previous studies [19, 22, 34], and no sample size calculation was performed, which may limit the application of the conclusion. This study showed that the screw-tightening methods significantly impacted the prevention of screw loosening in different-diameter implants in each system. Oral cyclic loading *in vivo* can be rather complicated, and other factors can influence screw loosening, such as temperature variations, changes in the pH of the liquid medium, and the variability of the load and angle imposed on the implants [8, 36]. Considering these limitations, long-term studies on the effectiveness of tightening method B in clinical situations using a larger number of samples and loading cycles are needed to verify the findings. The specific mechanism of screw loosening remains to be clarified in further studies in order to advance effective countermeasures in the clinic.

## 5. Conclusion

In summary, with 4.60–11.26 Ncm removal torque loss, the method with the preferable clinical operation time is torquing and then immediate loosening and retorquing, which increases the screw joint immediate stability by 6.28%−21.58% and the long-term stability by 11.77%−44.87% compared with torquing only once. This technique results in comparable screw joint immediate- and long-term stability compared with retorquing after 10 min and is recommended in clinical settings.

## Figures and Tables

**Table 1:** Specifications of the tested implant systems, abutment screws, implant abutments, and mean value ± standard deviation of IRT (Ncm), PRT (Ncm), and TLR (%) in each group.

System	Dimensions of implants (mm)	Screw	Initial removal torque (Ncm)			Difference of initial removal torque (%)		Postloading removal torque (Ncm)			Difference of postloading removal torque (%)		Removal torque loss (Ncm)			Removal torque loss rate (%)			Difference of removal torque loss rate (%)	
			A	B	C	(A − C)/C	(B − C)/C	A	B	C	(A−C)/C	(B−C)/C	A	B	C	A	B	C	(C−A)	(C−B)
Nobel	Ø3.5 × 11.5	NP	29.70 ± 0.47^Aa^	29.78 ± 0.66^Aa^	27.58 ± 0.54^Ab^	7.69%	7.99%	20.62 ± 0.47^Aa^	20.58 ± 0.79^Aa^	17.54 ± 0.85^Ab^	17.56%	17.33%	9.08 ± 0.65^Aa^	9.20 ± 0.54^Aab^	10.04 ± 0.47^Ab^	30.56 ± 1.89^Aa^	30.90 ± 1.84^Aa^	36.42 ± 2.07^Ab^	5.86%	5.52%
	Ø4.3 × 11.5	RP	28.42 ± 0.55^Ba^	28.56 ± 0.40^Ba^	26.42 ± 0.72^Bb^	7.57%	8.10%	17.90 ± 0.29^Ba^	18.00 ± 0.43^Ba^	15.38 ± 0.63^Bb^	16.38%	17.04%	10.48 ± 0.48^Ba^	10.56 ± 0.15^ABa^	11.04 ± 0.15^ABa^	37.01 ± 0.90^Ba^	36.98 ± 0.75^Ba^	41.80 ± 0.86^Bb^	4.79%	4.82%
	Ø5.0 × 11.5	RP	32.68 ± 0.48^Ca^	32.48 ± 0.56^Ca^	30.56 ± 0.69^Cb^	6.94%	6.28%	21.36 ± 0.54^Aa^	21.22 ± 0.61^Aa^	18.60 ± 0.85^Ab^	14.84%	14.09%	11.32 ± 0.26^Ca^	11.26 ± 0.23^Ba^	11.96 ± 0.17^Bb^	34.64 ± 0.93^Ca^	34.68 ± 0.94^Ca^	39.16 ± 1.40^Cb^	4.52%	4.48%

Straumann	Ø3.3 × 12.0	NC	30.00 ± 0.57^Aa^	29.82 ± 0.48^Aa^	27.72 ± 0.70^Ab^	8.23%	7.58%	24.28 ± 0.29^Aa^	24.12 ± 0.48^Aa^	21.58 ± 0.67^Ab^	12.51%	11.77%	5.72 ± 0.57^Aa^	5.70 ± 0.48^Aa^	6.14 ± 0.37^Aa^	19.05 ± 1.61^Aa^	19.11 ± 1.51^Aa^	22.15 ± 1.28^Ab^	3.10%	3.04%
	Ø4.1 × 12.0	RC	31.92 ± 0.33^Ba^	32.04 ± 0.36^Ba^	29.70 ± 0.66^Bb^	7.47%	7.88%	27.20 ± 0.38^Ba^	27.22 ± 0.32^Ba^	24.18 ± 0.68^Bb^	12.49%	12.57%	4.72 ± 0.16^ABa^	4.82 ± 0.28^Ba^	5.52 ± 0.22^ABb^	14.79 ± 0.55^Ba^	15.04 ± 0.79^Ba^	18.59 ± 0.81^Bb^	3.80%	3.55%
	Ø4.8 × 12.0	RC	34.12 ± 0.36^Ca^	33.96 ± 0.29^Ca^	31.62 ± 0.52^Cb^	7.91%	7.40%	29.86 ± 0.65^Ca^	29.60 ± 0.36^Ca^	26.38 ± 0.59^Cb^	13.19%	12.21%	4.26 ± 0.30^Ba^	4.36 ± 0.34^Ba^	5.24 ± 0.11^Bb^	12.49 ± 0.99^Ca^	12.84 ± 0.96^Ca^	16.58 ± 0.56^Cb^	4.09%	3.74%

WEGO	Ø3.4 × 11.0	NP	11.92 ± 1.04^Aa^	11.64 ± 0.57^Aa^	10.30 ± 0.41^Ab^	15.73%	13.01%	4.08 ± 0.77^Aa^	4.04 ± 0.40^Aa^	2.98 ± 0.26^Ab^	36.91%	35.57%	7.84 ± 0.71^Aa^	7.60 ± 0.23^Aa^	7.32 ± 0.20^ABa^	65.88 ± 4.83^Aa^	65.35 ± 1.95^Aa^	71.10 ± 1.58^Aa^	5.22%	5.75%
	Ø3.8 × 11.0	RP	14.76 ± 0.40^Ba^	14.08 ± 1.30^Ba^	12.14 ± 0.82^Bb^	21.58%	15.98%	6.78 ± 0.61^Ba^	6.52 ± 0.98^Ba^	4.68 ± 0.80^ABb^	44.87%	39.32%	7.98 ± 0.49^Aa^	7.56 ± 0.38^Aa^	7.46 ± 0.38^Aa^	54.09 ± 3.56^ABa^	53.89 ± 2.83^Ba^	61.63 ± 4.66^ABb^	7.54%	7.74%
	Ø4.3 × 11.0	RP	15.68 ± 0.76^BCa^	15.46 ± 0.93^BCa^	13.90 ± 0.78^Cb^	12.81%	11.22%	8.98 ± 0.91^Ca^	8.74 ± 0.90^Ca^	6.92 ± 0.79^BCb^	29.77%	26.30%	6.70 ± 0.28^Ba^	6.72 ± 0.49^ABa^	6.98 ± 0.19^Ba^	42.84 ± 3.26^Ba^	43.54 ± 3.26^Ca^	50.34 ± 2.98^BCb^	7.50%	6.80%
	Ø5.0 × 11.0	RP	16.82 ± 0.62^Ca^	16.38 ± 0.90^Ca^	15.18 ± 0.52^Db^	10.80%	7.91%	10.72 ± 0.93^Da^	10.46 ± 0.91^Da^	8.68 ± 0.61^Cb^	23.50%	20.51%	6.10 ± 0.41^Ba^	5.92 ± 0.58^Ba^	6.50 ± 0.12^Ca^	36.35 ± 3.49^Ba^	36.18 ± 3.34^Da^	42.87 ± 2.11^Cb^	6.52%	6.69%

Within the same column, mean values followed by the same capital letter are statistically similar (*P* > 0.05), whereas mean values followed by different capital letters are statistically different (*P* < 0.05) among different-diameter implants using the same tightening method. Within the same row, mean values followed by the same lowercase letter are statistically similar (*P* > 0.05), whereas mean values followed by different lowercase letters are statistically different (*P* < 0.05) among same-diameter implants using different tightening methods.

**Table 2:** The representative numbers for the comparisons.

Comparison number	Comparison			
Nos. 1–3	The IRT between different	Tightening methods	In group Nobel Ø3.5, Ø4.3, Ø5.0	
Nos. 4–6			In group Straumann Ø3.3, Ø4.1, Ø4.8	
Nos. 7–10			In group WEGO Ø3.4, Ø3.8, Ø4.3, Ø5.0	
Nos. 11–19		Diameters groups	In groups A, B, C	Nobel: nos. 11–13 Straumann: nos. 14–16 WEGO: nos. 17–19

Nos. 20–22	The PRT between different	Tightening methods	In group Nobel Ø3.5, Ø4.3, Ø5.0	
Nos. 23–25			In group Straumann Ø3.3, Ø4.1, Ø4.8	
Nos. 26–29			In group WEGO Ø3.4, Ø3.8, Ø4.3, Ø5.0	
Nos. 30–38		Diameters groups	In groups A, B, C	Nobel: nos. 30–32 Straumann: nos. 33–35 WEGO: nos. 36–38

Nos. 39–41	The removal torque loss between different	Tightening methods	In group Nobel Ø3.5, Ø4.3, Ø5.0	
Nos. 42–44			In group Straumann Ø3.3, Ø4.1, Ø4.8	
Nos. 45–48			In group WEGO Ø3.4, Ø3.8, Ø4.3, Ø5.0	
Nos. 49–57		Diameters groups	In groups A, B, C	Nobel: nos. 49–51 Straumann: nos. 52–54 WEGO: nos. 55–57

Nos. 58–60	The TLR between different	Tightening methods	In group Nobel Ø3.5, Ø4.3, Ø5.0	
Nos. 61–63			In group Straumann Ø3.3, Ø4.1, Ø4.8	
Nos. 64–67			In group WEGO Ø3.4, Ø3.8, Ø4.3, Ø5.0	
Nos. 68–76		Diameters groups	In group A, B, C	Nobel: nos. 68–70 Straumann: nos. 71–73 WEGO: nos. 74–76

Nos. 77–79	The operating time between different tightening methods in Nobel (no.77), Straumann (no.78), WEGO (no.79)			

**Table 3:** The operating time (s) of three different tightening methods in each system.

Tightening method	System		
	Nobel	Straumann	WEGO
A	626.65 ± 1.68^a^	623.94 ± 1.43^a^	616.86 ± 0.97^a^
B	29.46 ± 1.47^b^	28.47 ± 1.42^b^	18.79 ± 1.15^b^
C	21.34 ± 1.63^c^	19.04 ± 1.12^c^	11.66 ± 0.78^c^

Within the same column, mean values followed by the same letter are statistically similar (*P* > 0.05), mean values followed by different letters are statistically different (*P* < 0.05) in each implant system using different tightening methods.

## Data Availability

The data that support the findings of this study are available from the corresponding author upon reasonable request.
